# Changes in Physical Activity and Psychological Status in Patients with COPD Between Pre- and Post-COVID-19 Assessments: A Pilot Observational Longitudinal Cohort Study

**DOI:** 10.3390/healthcare14193142

**Published:** 2026-09-22

**Authors:** Beatriz Enrich Alvarez, Marta Ortega Bravo, Eva Artigues Barberà, Leonardo Machado, Assumpta Ensenyat, Pere Godoy Garcia, Joaquim Sol

**Affiliations:** 1 Cappont Primary Care Centre, Lleida, Institut Català de la Salut (ICS), 08007 Lleida, Spain; benrich.lleida.ics@gencat.cat; 2 Fundació Institut Universitari per la Recerca a l’Atenció Primària Jordi Gol i Gurina (IDIAPJGol), 08007 Barcelona, Spain; 3 Department of Medicine, Faculty of Medicine, University of Lleida, 25003 Lleida, Spain; 4 Almacelles Primary Care Centre, Lleida, Institut Català de la Salut (ICS), 08007 Lleida, Spain; 5 Balàfia-Pardinyes-Secà de Sant Pere Primary Care Centre, Lleida, Institut Català de la Salut (ICS), 08007 Lleida, Spain; 6 Research Support Unit Lleida, Fundació Institut Universitari per la Recerca a l’Atenció Primària Jordi Gol i Gurina (IDIAPJGol), 08007 Lleida, Spain; 7 Complex System Research Group (SGR 975), Institut Nacional d’Educació Física de Catalunya (INEFC), University of Lleida, 25003 Lleida, Spain; 8 Lleida Biochemical Research Institute (IRBLleida), 25003 Lleida, Spain; 9 CIBER of Epidemiology and Public Health, Instituto de Salud Carlos III, 28029 Madrid, Spain; 10 Metabolic Physiopathology Research Group, Experimental Medicine Department, University of Lleida—Lleida Biochemical Research Institute (UdL-IRBLleida), 25198 Lleida, Spain

**Keywords:** chronic obstructive pulmonary disease, COPD, physical activity, sedentary behavior, accelerometry, COVID-19 pandemic, primary care, anxiety

## Abstract

**Highlights:**

**What are the main findings?**

- Patients with COPD showed a predominantly sedentary physical activity profile, with very low levels of moderate-to-vigorous physical activity.
- Daily physical activity was lower at the post-COVID-19 assessment, particularly in terms of daily steps, while time spent in sedentary mode increased.
- Anxiety symptoms and perceived health status showed less favorable profiles at the post-COVID-19 assessment, while depressive symptoms remained relatively stable.

**What are the implications of the main findings?**

- When developing management strategies for patients with COPD, physical activity and sedentary behavior should be considered in addition to psychological well-being and perceived health status.
- Monitoring these dimensions may help identify patients with persistent inactivity or poorer health status, although larger controlled longitudinal studies are needed to confirm these preliminary observations.

**Abstract:**

**Background/Objectives**: Physical activity is a key prognostic factor in chronic obstructive pulmonary disease (COPD), but changes in physical activity patterns during the COVID-19 pandemic remain insufficiently characterized. **Methods**: We conducted an observational longitudinal cohort study in patients with COPD managed in primary care in Lleida, Spain. Baseline assessment was performed between December 2018 and February 2019 (pre-COVID-19 pandemic), and follow-up assessment was conducted between March and June 2021 (post-COVID-19 pandemic). Physical activity was measured using triaxial accelerometry, and clinical, functional, and psychological variables were collected. **Results**: Of the 192 screened patients, 13 patients were included in the baseline pre-COVID-19 cohort, of whom 7 completed the longitudinal follow-up assessment in the post-COVID-19 period. Among the seven patients with longitudinal follow-up, at baseline, participants exhibited a predominantly sedentary profile, spending approximately 80% of their time in a sedentary mode and accumulating a mean of 6179 steps/day. In the post-COVID-19 period, sedentary time increased to 85.7% and mean steps/day decreased to 3038. Patients also showed a worse psychological profile, mainly driven by increased anxiety levels. Exploratory analyses suggested variable associations between physical activity and clinical, health-related quality of life, and psychological measures. **Conclusions**: In this small exploratory longitudinal cohort, lower physical activity and a less favorable psychological profile, particularly regarding anxiety, were observed at the post-COVID-19 assessment compared with the baseline. Given the small, paired sample and observational design, these findings should be considered exploratory and hypothesis-generating. Further studies with larger controlled longitudinal cohorts are warranted to confirm these observations.

## 1. Introduction

Chronic obstructive pulmonary disease (COPD) is a heterogeneous respiratory condition characterized by persistent airflow limitation and respiratory symptoms [1]. Reduced physical activity (PA) is a common feature of COPD and is associated with symptom burden, risk of exacerbation, and mortality [2,3,4,5]. Reduced PA in COPD is multifactorial and may be related to dyspnea, dynamic hyperinflation, impaired gas exchange, peripheral muscle dysfunction, and deconditioning [6,7,8,9,10,11]. PA is therefore an important, potentially modifiable target in COPD management, and it is promoted as part of pulmonary rehabilitation and long-term disease management strategies [12,13,14,15]. 

The COVID-19 pandemic and mobility restrictions implemented to limit viral transmission substantially altered daily routines and physical activity patterns in the general population [16,17]. Patients with COPD may have been particularly vulnerable to these changes because of their pre-existing limitations in physical activity and their increased susceptibility to adverse health outcomes. Previous studies have reported reduced physical activity, increased sedentary behavior, and changes in health-related quality of life among patients with COPD during the COVID-19 pandemic [18,19].

However, important uncertainties remain regarding longitudinal changes in objectively measured physical activity in patients with COPD assessed before and after pandemic-related restrictions. Previous studies have provided important evidence of changes in physical activity and behavior during the COVID-19 pandemic [18,19]; however, longitudinal comparisons based on objective activity measurements obtained before the pandemic and reassessed after major restrictions remain limited. In addition, the relationships between physical activity and clinical, symptom-related, and psychological characteristics in this context have not been characterized sufficiently. Addressing these questions may help to generate hypotheses regarding longer-term changes in physical activity and psychological status in patients with COPD.

Therefore, we conducted an observational longitudinal cohort study in patients with COPD managed in primary care. The primary objective was to explore longitudinal changes in objectively measured physical activity among patients with paired pre- and post-COVID-19 assessments (*n* = 7). Secondary exploratory objectives were to describe changes in sedentary behavior, clinical symptoms, psychological status, and health-related quality of life, and to explore their potential associations with physical activity. Given the small longitudinal sample and observational design, these analyses were considered exploratory and hypothesis-generating, and were not designed to establish causal effects of the COVID-19 pandemic or COVID-19-related mobility restrictions.

## 2. Materials and Methods

### 2.1. Study Design

We assessed physical activity (PA) in patients with COPD during pre- and post-COVID-19 assessment periods and explored longitudinal changes between these two time points. The original study was a prospective observational study conducted in patients with COPD attending the Primary Care Health Center in Cappont de Lleida. Its primary objective was to assess physical activity levels in patients with COPD and the associated relationships with adverse events and quality of life. The planned sample size for the original prospective study was 123 participants; however, this recruitment target was not reached. Recruitment and baseline assessments were conducted between December 2018 and February 2019. Following the onset of the COVID-19 pandemic, a subset of participants from the original pre-COVID-19 analytical cohort was reassessed between March and June 2021 to explore changes in physical activity and clinical characteristics following COVID-19-related mobility restrictions. This longitudinal reassessment was not part of the original study design or sample-size calculation and was therefore considered exploratory.

### 2.2. Participants

Eligible patients were aged ≥ 40 years and had a diagnosis of COPD defined by a smoking history of ≥10 pack-years, a post-bronchodilator FEV1/FVC ≤ 0.7, and persistent respiratory symptoms under outpatient follow-up in primary care. To ensure diagnostic accuracy, only patients with spirometry performed within the previous year were considered eligible; those without a recent spirometry assessment were excluded according to the study protocol. Additional exclusion criteria included language barriers, inability to attend the study center, active neoplastic disease or follow-up by palliative care units, and clinically significant comorbidities (cardiovascular, metabolic, neurological, or musculoskeletal) that could limit physical activity.

Patients who declined participation after invitation were recorded as non-participants. Among those initially included in the cohort, withdrawals during the study period were defined as patients who died, could not be contacted for follow-up, or declined further participation. Participants without an available post-COVID-19 physical activity assessment were not included in the longitudinal paired analysis.

For the longitudinal analysis, only participants with physical activity measurements at both the pre-COVID-19 and post-COVID-19 assessments were included. Therefore, the longitudinal analysis was limited to seven participants with paired measurements at both time points.

#### Participant Flow

A total of 192 patients with COPD and a history of smoking aged > 40 years were initially screened. Of these, 164 patients were excluded because more than a year had elapsed since their last spirometry evaluation, beyond the annual spirometric follow-up interval recommended by GOLD [1]. The remaining 28 patients met the inclusion criteria and were invited to participate; 6 declined, leaving 22 eligible individuals. Of the 22 participants who initially agreed to participate, 8 completed the initial interview but did not attend the subsequent visit for accelerometer placement. One additional participant completed accelerometer monitoring but experienced a COPD exacerbation during the recording period; therefore, this accelerometry assessment was considered invalid for evaluating habitual physical activity in a clinically stable condition and was excluded. Consequently, 13 participants had valid pre-COVID-19 accelerometry data and constituted the baseline analytical cohort. Of these, 6 were further excluded (1 died, 3 were not reachable by phone, and 2 declined participation), yielding a final post-COVID-19 study population of 7 patients (Figure 1).

### 2.3. Variables and Measurements

The following variables were included in the data collection notebook (Supplementary Files S1–S5):

- Demographic and clinical variables, including age, sex, smoking, household, and employment status; exacerbations and hospital admissions in the previous year; current inhaled treatment for COPD (SABA, SAMA, LABA, LAMA, LABA + LAMA, LABA + ICS, LABA + LAMA + ICS); supplemental oxygen therapy; and comorbidity burden assessed using the age-adjusted Charlson Comorbidity Index (CCI) [20] (File S1).
- Airflow obstruction based on spirometry performed within the previous year (FEV1).
- Dyspnea severity assessed using the modified Medical Research Council (mMRC) scale and disease impact assessed using the COPD Assessment Test (CAT), both validated for use in Spanish [21,22] (Files S2 and S3).
- Anxiety and Depression assessed using the Hospital Anxiety and Depression Scale (HADS), validated for use in Spanish [23,24] (File S4).
- Quality of life assessed using the EQ-5D score, validated for use in Spanish [25] (File S5).
- Classification of COPD using the GOLD 2017 scale in the ABCD subclasses and the GOLD 2025 framework in the ABE subclasses [1,26].
- Physical activity was objectively assessed using triaxial accelerometers (ActiGraph GT3X, Pensacola, FL, USA). Participants were instructed to wear the accelerometer at the waist using an elastic belt for at least 7 consecutive days, including weekend days. Accelerometers were configured at a sampling frequency of 100 Hz, and data were aggregated into 60 s epochs for analysis using the ActiLife 6.0 software (ActiGraph, Pensacola, FL, USA). A valid accelerometry assessment required at least 4 valid recording days, including at least 1 weekend day, with a minimum wear time of 8 h/day. Non-wear time was identified according to the Troiano 2008 criterion [27], defined as ≥60 consecutive minutes of zero counts, allowing up to 2 min with values ≤ 100 counts/min. Sleep periods were excluded from the physical activity analysis. Intervals with no recorded accelerometry data were treated as missing and were not included in the analysis. The same acquisition and processing criteria were applied at both assessment periods.

Overall activity was quantified using vector magnitude (VM), expressed as counts per minute (CPM), with the percentage of analyzed time spent at different physical activity intensities and steps/day. Sedentary behavior was defined as <100 CPM, light-intensity physical activity (LPA) as 100–2019 CPM, and moderate-to-vigorous physical activity (MVPA) as ≥2020 CPM [27]. Sedentary behavior was also characterized by total sedentary time and the number and duration of daily sedentary bouts. These thresholds were applied consistently across both assessment periods and were considered standardized operational definitions of activity intensity rather than absolute physiological thresholds. The analysis was complemented by participants’ daily activity diaries collected during the 7-day assessment period [28].

Classification of PA trends according to the activity clusters described by Tudor-Locke [29].

### 2.4. Ethical Considerations

The ACTIV-POC study (protocol code P18/115) was reviewed and approved by the Clinical Research Ethics Committee of IDIAP Jordi Gol. The initial project was evaluated at a Committee meeting on 26 September 2018, with the corresponding certificate dated 12 February 2019. Subsequent amendments were reviewed in August 2020 and February 2021. All participants provided written informed consent before participation, and were informed that participation was voluntary and that they could withdraw from the study at any time without affecting their medical care.

### 2.5. Statistical Analysis

Data were coded and analyzed using the RStudio v. 2025.05 statistical software. Continuous variables are summarized using means and standard deviations (SDs) or medians and interquartile ranges (IQRs), as appropriate. Categorical variables are represented as absolute and relative frequencies. We first described all participants included in the pre-COVID-19 assessment and then those who participated in both the pre- and post-COVID-19 assessments. For the longitudinal analysis, only participants with physical activity measurements at both assessment time points were included. Given the small sample size and paired observations, changes in sedentary time, light-intensity physical activity (LPA), moderate-to-vigorous physical activity (MVPA), vector magnitude counts per minute (CPM), and steps/day were assessed using the exact two-sided Wilcoxon signed-rank test for paired samples. Continuous variables in the longitudinal analysis are summarized using mean ± SD and median (IQR). Spearman’s rank correlation coefficient was used to explore associations between physical activity measures and clinical, health-related quality of life, and psychological variables.

Given the small, paired sample size (*n* = 7), all longitudinal inferential and correlation analyses were considered exploratory and hypothesis-generating. No adjustment for multiple comparisons was applied to the correlation analyses. Exact *p*-values are reported for completeness; however, individual *p*-values and correlation coefficients should not be interpreted as confirmatory evidence given the limited statistical power, multiple pairwise comparisons included, and exploratory nature of the analyses.

## 3. Results

### 3.1. Demographic and Clinical Characteristics

All participant characteristics are presented in Table 1. The pre-COVID-19 cohort included 13 participants. Of these, seven participants completed physical activity measurements at both the pre-COVID-19 and post-COVID-19 assessments and therefore constituted the longitudinal paired cohort.

The baseline pre-COVID-19 cohort included 12 men and 1 woman, with a mean age of 67.47 years (SD 9.66). All participants lived in an urban area, 11 lived with others, and 6 were active smokers. The mean FEV1 was 55.07% (range, 13–90%). Six participants (46.15%) had experienced two or more moderate/severe exacerbations during the previous year.

The longitudinal paired cohort comprised seven participants. At the pre-COVID-19 assessment, these participants had a mean age of 70.51 years (SD 11.23); six were men, and one was a woman. Five participants lived with others, and four were active smokers. The mean FEV1 was 47.28% predicted (range, 13–90%). Two participants (28.57%) experienced two or more moderate/severe exacerbations during the previous year, none requiring hospital admission. At the post-COVID-19 assessment, the mean age was 72.73 years (SD 11.22); six participants were men, and one was a woman; six lived with others; and three were active smokers. The mean FEV1 remained 47.28% predicted (range, 13–90%). Spirometry was not repeated at the post-COVID-19 assessment; therefore, the baseline spirometric values were retained for descriptive purposes. One participant (14.28%) experienced a severe exacerbation requiring hospital admission during the follow-up period.

Of the 13 participants assessed at baseline, 7 (53.8%) completed the longitudinal follow-up and 6 (46.2%) did not. Baseline characteristics of completers and non-completers are presented in Supplementary Table S1. Descriptively, non-completers were younger and had higher FEV_1_, lower CAT and mMRC scores, and a higher proportion of participants with ≥2 exacerbations and GOLD E classification. Baseline daily step counts were similar between groups. Given the very small group sizes, no inferential statistical comparisons were made.

Clinical indices for the pre-COVID-19 and post-COVID-19 assessments are summarized in Table 2. In the full pre-COVID-19 cohort, the mean CAT score was 12.23 (SD 8.58; range, 1–30), and 61.5% of participants experienced some degree of dyspnea (mMRC ≥ 1). Within the longitudinal paired cohort, the mean CAT score was 13.42 (SD 10.58; range, 1–30) at the pre-COVID-19 assessment and 13.57 (SD 10.03; range, 0–25) at the post-COVID-19 assessment. Mean mMRC increased from 1.00 (SD 0.81; range, 0–2) to 1.57 (SD 1.27; range, 0–4) in the longitudinal paired cohort.

GOLD classification was assessed descriptively among the seven participants using paired assessments. Based on the GOLD 2017 classification, the pre-COVID-19 assessment included three patients in group A, two in group B, two in group D, and none in group C. When the same patients were reclassified according to the GOLD 2025 framework, three were classified as group A, two as group B, and two as group E during the pre-COVID-19 assessment. At post-COVID-19 follow-up, two patients were classified as group A, four as group B, and one as group E. Given the small, paired sample, these changes are descriptive and should not be considered representative of population-level alterations in disease classification. The GOLD classification trajectories (GOLD 2017 at pre-COVID-19 assessment, reclassification according to GOLD 2025 at pre-COVID-19 assessment, and GOLD 2025 at post-COVID-19 assessment) for the seven participants with paired assessments are presented in Supplementary Table S2.

Health-related quality of life assessed using the EQ-5D showed lower mean scores in the post-COVID-19 assessment in the longitudinal paired cohort. The mean EQ-5D utility index decreased from 0.749 (SD 0.207) at the pre-COVID-19 assessment to 0.642 (SD 0.216) at the post-COVID-19 assessment. Similarly, the mean EQ-VAS score decreased slightly from 60.00 (SD 22.36) to 57.14 (SD 19.97).

For individual EQ-5D dimensions, mobility-related problems were reported by 42.9% of participants at pre-COVID-19 and 85.71% at post-COVID-19 assessments. Pain/discomfort increased from 28.6% to 57.1%, anxiety/depression from 57.1% to 71.4%, and self-care from 14.3% to 28.6%. In contrast, problems with usual activities decreased from 28.6% to 14.3%. Given the small, paired sample, these changes are descriptive and should be interpreted cautiously.

### 3.2. Physical Activity Patterns

Physical activity was assessed longitudinally in the seven participants using paired accelerometry data for both assessments. The pre-COVID-19 assessment included a mean of 7.29 recording days (SD 0.49) and a mean recorded time of 9591.43 min (SD 702.65). All participants in the post-COVID-19 assessment had 8 recording days, with a mean recorded time of 7755.0 min (SD 1638.8). All seven participants met the predefined accelerometry validity criteria at both assessments.

For the pre-COVID-19 assessment, participants spent 82.07% (SD 6.38) of the mean recorded time in a sedentary mode, 15.92% (SD 4.67) in light-intensity physical activity (LPA), and 2.01% (SD 2.20) in moderate-to-vigorous physical activity (MVPA). For the post-COVID-19 assessment, sedentary time increased to 85.66% (SD 9.19), while LPA and MVPA decreased to 13.17% (SD 8.16) and 1.19% (SD 1.13), respectively. Mean steps/day decreased from 6179.82 (SD 3862.65) at the pre-COVID-19 assessment to 3038.05 (SD 1914.70) at the post-COVID-19 assessment, corresponding to a mean decrease of 3141.77 steps/day. Mean vector magnitudes were 286.89 CPM (SD 144.42) and 312.61 CPM (SD 249.45) for the pre- and post-COVID-19 assessments, respectively. For daily step count, the corresponding median values were 5348.43 (IQR 3474.50–8595.00) and 2584.63 (IQR 1432.31–4166.38) steps/day for the pre- and post-COVID-19 assessments, respectively. The median paired change was −2272.71 steps/day (95% CI: −5481.54 to −805.38; exact Wilcoxon *p* = 0.031; rank–biserial correlation = −0.93). Changes in sedentary time, LPA, MVPA, and vector magnitude were smaller and more variable. Complete descriptive statistics, paired changes, confidence intervals, effect sizes, and exact Wilcoxon test results are presented in Table 3. Given the small, paired sample (*n* = 7), these longitudinal comparisons should be considered exploratory.

Based on the predefined steps/day categories, four of the seven participants achieved ≥5000 steps/day at the pre-COVID-19 assessment, including two participants who exceeded 10,000 steps/day. At the post-COVID-19 assessment, only one participant achieved ≥5000 steps/day, while six participants reached <5000 steps/day and were therefore classified as sedentary according to the predefined categories. Individual steps/day and changes between assessments are presented in Table 4. Six of the seven participants decreased in steps/day between assessments, whereas one participant increased by 456.75 steps/day. Three participants changed from non-sedentary to sedentary categories, three remained sedentary at both assessments, and one remained non-sedentary at both assessments. The largest decrease was observed in one participant, whose steps/day decreased by 9613.66, from 11,191.29 to 1577.63 steps/day. Individual trajectories of physical activity measures between the pre- and post-COVID-19 assessments are shown in Supplementary Figure S1. Overall, the individual trajectories showed substantial inter-individual variability in the magnitude and direction of change. Changes in sedentary time, light-intensity physical activity, moderate-to-vigorous physical activity, and vector magnitude were more heterogeneous across participants. These individual trajectories complement the group-level analyses by illustrating the variability underlying the observed changes in physical activity measures. 

### 3.3. Exploratory Associations Between Clinical Variables and Physical Activity

Exploratory analyses of the seven participants from paired pre- and post-COVID-19 assessments showed variable associations between physical activity measures and clinical, health-related quality of life, and psychological variables. At the pre-COVID-19 assessment, higher physical activity, particularly steps/day and vector magnitude, was inversely associated with CAT scores, and higher steps/day and vector magnitude were positively associated with EQ-VAS scores. Exploratory associations were also observed between several physical activity measures and the Charlson Comorbidity Index. Anxiety symptoms showed variable associations with physical activity, including an inverse association with sedentary time and a positive association with light-intensity physical activity. Associations with depressive symptoms were generally small in magnitude and inconsistent across physical activity measures. 

At the post-COVID-19 assessment, higher steps/day showed inverse associations with CAT and mMRC scores, whereas associations between physical activity and EQ-5D measures did not show a consistent pattern. In particular, no consistent association was observed between physical activity measures and the EQ-5D utility index. Associations with psychological and comorbidity measures were variable across physical activity measures and assessment periods. Given the very small, paired sample (*n* = 7), all correlations are exploratory and should not be interpreted as robust or definitive relationships (Figure 2 and Figure 3).

### 3.4. Psychological Status

Psychological assessment using the Hospital Anxiety and Depression Scale (HADS) showed a modest increase in the mean total HADS score from 11.71 ± 8.63 in the pre-COVID-19 period to 13.43 ± 10.49 in the post-COVID-19 period. The proportion of participants classified within the clinical range (≥11) remained unchanged at 42.9% at both assessments. For anxiety, the mean HADS-A score increased from 5.14 ± 5.17 to 7.57 ± 5.19. The proportion of participants within the normal range decreased from 71.4% to 42.9%, and the proportion classified as borderline increased from 0% to 28.6%; the proportion in the clinical range remained at 28.6% for both assessments. In contrast, mean depressive symptoms, assessed by HADS-D, were slightly lower for the post-COVID-19 assessment (6.60 ± 5.20 vs. 5.86 ± 6.34); the proportion of participants in the clinical range decreased from 28.6% to 14.3%. Given the small, paired sample (*n* = 7), these findings should be interpreted cautiously as they are descriptive rather than evidence of a definitive change in psychological status. HADS results are summarized in Table 5.

## 4. Discussion

In this study, we described the demographic and clinical characteristics of patients with COPD managed in primary care in Lleida and examined physical activity patterns at pre- and post-COVID-19 assessments. The study population consisted predominantly of men who were overweight, active or former smokers, and had moderate to severe airflow obstruction, broadly consistent with previous Spanish COPD cohorts [30,31,32].

A predominantly sedentary behavioral profile was observed in our cohort. Among the seven participants with complete paired accelerometry data, the pre-COVID assessment identified that approximately 82% of recorded time was spent in a sedentary mode. By contrast, moderate-to-vigorous physical activity accounted for only 2% of recorded time. Mean daily step count was approximately 6180 steps/day, although substantial inter-individual variability was observed. These findings are consistent with previous studies showing markedly reduced physical activity in patients with COPD compared with healthy controls [10,11]. Given the established association between lower physical activity and poorer outcomes in COPD [5,32], the high sedentary burden observed in this cohort may have relevant clinical implications.

Exploratory correlation analyses suggested relationships between physical activity and respiratory symptom burden; however, these patterns varied with the measure and assessment period. For the pre-COVID-19 assessment, higher physical activity, particularly steps/day and vector magnitude, was associated with lower CAT scores; for the post-COVID-19 assessment, higher steps/day showed inverse associations with both CAT and mMRC scores. Exploratory associations were also observed between the Charlson Comorbidity Index and several physical activity measures at the pre-COVID-19 assessment, including more sedentary behavior and lower scores for light-intensity physical activity and vector magnitude. However, these patterns were not observed consistently in the post-COVID-19 assessment. Associations involving the age-adjusted Charlson Index were generally inconsistent across physical activity measures. Given that these correlations were based on only seven participants, they should be regarded as exploratory and potentially unstable rather than evidence of robust relationships. In addition, because the eligibility criteria excluded patients with significant cardiovascular, metabolic, neurological, or musculoskeletal comorbidities, the range of comorbidities was restricted. This may have influenced the observed associations. Their generalizability to the broader COPD population with multimorbidity is also limited.

From a physiological perspective, baseline FEV1 was not clearly associated with physical activity in our cohort, in agreement with previous studies [7,8,33]. Spirometry was not repeated at the post-COVID-19 assessment due to restrictions on pulmonary function testing during the pandemic; therefore, longitudinal relationships between changes in FEV1 and physical activity could not be evaluated. Other respiratory mechanical parameters, such as air trapping and dynamic hyperinflation, are reportedly more closely related to daily activity levels [34,35,36,37,38,39,40,41,42,43]. Thus, airflow obstruction alone may not fully explain differences in daily physical activity, with symptoms and functional limitations as additional relevant factors.

In the post-COVID-19 assessment period, all participants were receiving bronchodilator therapy, whereas one participant in the pre-COVID-19 cohort did not. Although this may reflect differences in treatment coverage over time, therapeutic adequacy cannot be inferred from these data and should be interpreted in the context of the small sample and changes in clinical management. Pharmacological treatment may influence symptom control and, consequently, physical activity patterns [36,37,38,39,40]. More broadly, differences or changes in treatment, smoking status, comorbidity burden, exacerbations, hospitalization, symptoms and dyspnea, and other time-dependent clinical factors may have influenced physical activity independently of the pandemic context. Oxygen therapy remained unchanged, and no participant underwent pulmonary rehabilitation during follow-up. Nevertheless, the very small longitudinal sample precluded reliable adjustment for the potential influence of these clinical factors.

The distribution of GOLD categories also differed between assessment periods. Among the seven participants with paired assessments, two were classified as GOLD D according to the 2017 classification at the pre-COVID-19 assessment. When reclassified according to the GOLD 2025 framework, these two participants were GOLD E. At the post-COVID-19 assessment, one participant remained in GOLD E, while the number classified as GOLD B increased from two to four. These descriptive changes should be interpreted cautiously, given the very small, paired sample and the differences between the GOLD classification frameworks. Importantly, changes in GOLD categories should not be interpreted as changes in COPD’s physiological severity. Spirometry was not repeated at the post-COVID-19 assessment; therefore, longitudinal changes in airflow obstruction could not be evaluated. The observed redistribution across GOLD categories should be understood as a descriptive change in classification based on the available symptom and exacerbation information. The pattern of exacerbations also differed between assessment periods; however, the observed redistribution of GOLD categories cannot be attributed specifically to changes in exacerbation history. Public health measures during the pandemic, including social distancing and facemask use, have been associated with reductions in COPD exacerbations and related admissions in other settings [1,41,42,43]. However, no causal relationship between these measures, exacerbation patterns, and the changes in GOLD classification was established in our cohort.

The most notable longitudinal change in physical activity was the reduction in daily step count. Among the seven paired participants, mean steps/day decreased from 6179.82 at the pre-COVID-19 assessment to 3038.05 at the post-COVID-19 assessment. This corresponds to a mean reduction of 3141.77 steps/day (approximately 51%). The median paired reduction was 2272.71 steps/day, with individual changes ranging from a decrease of 9613.66 steps/day to an increase of 456.75 steps/day. The exact Wilcoxon signed-rank analysis yielded a *p*-value of 0.031 for this comparison. Changes in sedentary time, light physical activity, moderate-to-vigorous physical activity, and vector magnitude showed smaller and more variable patterns. Given the small, paired sample, these analyses are exploratory, and the *p*-values should not be interpreted as definitive evidence of longitudinal change.

Previous longitudinal studies have described gradual reductions in daily step count in patients with COPD, including an estimated decline of approximately 300 steps/day/year [32]. Reductions in physical activity have also been reported in relation to exacerbated COPD [44]. In the present cohort, six of the seven participants achieved fewer daily steps at the post-COVID-19 assessment, while one participant increased their step count. Although the magnitude of the observed reduction was substantial, the absence of a contemporaneous control group prevents distinguishing potential pandemic-related effects from other variables, such as natural disease progression, aging, exacerbations, seasonal variation, treatment changes, or other time-dependent factors. Nevertheless, the observed pattern is consistent with previous reports of reduced physical activity among patients with COPD during and following pandemic-related restrictions [17,19].

Psychological assessment showed a less favorable anxiety profile at the post-COVID-19 assessment. Mean HADS-A increased from 5.14 ± 5.17 to 7.57 ± 5.19, and was accompanied by a reduction in the proportion of participants within the normal range and an increase in the borderline category. However, the proportion classified within the clinical range remained unchanged at 28.6%. In contrast, depressive symptoms remained relatively stable, with mean HADS-D decreasing slightly from 6.60 ± 5.20 to 5.86 ± 6.34 and the proportion of patients in the clinical range decreasing from 28.6% to 14.3%. Exploratory correlations between anxiety symptoms and physical activity were observed at the pre-COVID-19 assessment, including associations with sedentary time and light-intensity physical activity; however, these patterns were not consistently reproduced in the post-COVID-19 assessment. Associations involving depressive symptoms were not consistent across physical activity measures. Therefore, no directional, mediating, or causal relationship was inferred between psychological status and physical activity data.

Health-related quality of life also showed a less favorable overall profile at the post-COVID-19 assessment. The mean EQ-5D utility index decreased from 0.749 to 0.642, while the EQ-VAS decreased slightly from 60.0 to 57.14. A higher proportion of participants reported problems with mobility, pain/discomfort, self-care, and anxiety/depression at the post-COVID-19 assessment, whereas problems with usual activities were reported less frequently. These findings suggest a heterogeneous pattern across individual EQ-5D dimensions rather than a uniform deterioration in all aspects of health-related quality of life. Exploratory analyses further showed that higher physical activity was associated with better self-perceived health on the EQ-VAS during the pre-COVID-19 assessment. By contrast, these associations were not consistently maintained at the post-COVID-19 assessment. In particular, the EQ-5D utility index showed minimal association with physical activity measures at the post-COVID-19 assessment. Given the small, paired sample, these findings should be interpreted cautiously.

Taken together, the findings suggest that longitudinal changes observed in physical activity, psychological status, and health-related quality of life cannot be attributed to a single factor. Lower daily step counts for the post-COVID-19 assessment occurred alongside a higher proportion of sedentary behavior and descriptive changes in respiratory symptoms, anxiety, and perceived health status. However, these measures did not show uniform or consistently parallel changes. Sedentary behavior and physical activity are distinct but interrelated dimensions [10,45,46]. Importantly, the observational design, small, paired sample, and absence of a contemporaneous control group prevent attributing these changes specifically to COVID-19-related mobility restrictions or other pandemic-related factors.

From a clinical perspective, these preliminary observations highlight the potential value of considering physical activity and sedentary behavior alongside respiratory symptoms, psychological well-being, and perceived health status in patients with COPD. Monitoring these dimensions may help identify patients with persistent inactivity or poorer health status and inform individualized management strategies. However, the present study does not provide evidence for the effectiveness of any specific intervention. Larger controlled longitudinal studies are required to confirm these observations and determine whether interventions targeting physical activity, sedentary behavior, or psychological well-being translate into improved clinical outcomes.

### Limitations

This study has several limitations. First, the sample size was small, particularly for the longitudinal analyses, which included only seven participants with complete paired pre- and post-COVID-19 assessments. In addition, 9 of the 22 participants who initially agreed to participate did not contribute valid pre-COVID-19 accelerometry data; in particular, 8 did not attend the accelerometer placement visit and 1 experienced exacerbated COPD during the recording period. This rendered the accelerometry assessment unsuitable for evaluating habitual physical activity in a clinically stable condition. These baseline exclusions may have introduced selection bias. Furthermore, 6 of the 13 participants assessed at baseline did not complete longitudinal follow-up, representing substantial attrition. Baseline characteristics showed descriptive differences between completers and non-completers, although daily step counts were similar. Because reasons for loss to follow-up included death, inability to contact participants, and withdrawal, selection and survivor bias cannot be excluded. Consequently, the seven participants with paired assessments may not fully represent the original baseline cohort.

Second, the study was conducted in a single primary care setting and included a predominantly male population, which may limit external validity. In addition, the eligibility criteria excluded patients with significant cardiovascular, metabolic, neurological, or musculoskeletal comorbidities, resulting in a relatively selective population with a restricted spectrum of comorbidity. This may further limit the generalizability of the findings, particularly to patients with COPD who experience a greater burden of multimorbidity. The full pre-COVID-19 cohort (*n* = 13) and the paired longitudinal cohort (*n* = 7) should also be considered separately when interpreting the results.

Third, the observational design and the absence of a contemporaneous control group prevent causal inference. The observed changes in physical activity cannot be attributed specifically to the COVID-19 pandemic or mobility restrictions; they may also reflect aging, potential disease progression, changes in smoking status or pharmacological treatment, exacerbations, changes in dyspnea and symptom burden, pulmonary rehabilitation, social circumstances, or other time-dependent factors. Information on SARS-CoV-2 infection, post-COVID pandemic symptoms, vaccination status, and the intensity of mobility restrictions was not systematically collected at the individual level and therefore could not be accounted for in the analyses. Furthermore, changes in healthcare-seeking behavior and access during the pandemic may have influenced COPD exacerbations being identified and documented; therefore, potential detection or ascertainment bias cannot be excluded when interpreting changes in GOLD classification. These factors also represent potential sources of unmeasured confounding.

Fourth, the correlation analyses only included seven paired participants and involved multiple pairwise comparisons among several related measures. No adjustment was applied for multiple comparisons because these analyses were exploratory. In addition, sedentary time, LPA, and MVPA represent compositional components of recorded time, while steps/day and vector magnitude are derived from the same accelerometer signal; therefore, these physical activity measures are not statistically independent. The observed associations are consequently potentially unstable and should be considered hypothesis-generating rather than robust or definitive. Individual correlation coefficients and associated *p*-values are not confirmatory evidence of independent associations. Similarly, changes in HADS and EQ-5D should be interpreted cautiously given the small sample size. Changes in GOLD classification are descriptive and should not be interpreted as evidence of changes in physiological disease severity.

Finally, although accelerometry provides an objective assessment of physical activity, it does not capture the context or reasons behind underlying changes in activity. Standardized validity and processing criteria were applied consistently at both assessment periods, including minimum wear-time requirements, non-wear identification, and exclusion of sleep periods. Nevertheless, recorded time differed between the pre- and post-COVID-19 assessments, which may have introduced additional variability into the physical activity estimates despite all paired participants meeting the predefined accelerometry validity criteria. Although both weekday and weekend activity were represented in both assessments, differences in recording duration may have varied their relative contribution. Seasonality is another potential limitation. Pre-COVID-19 assessments were conducted between December 2018 and 1 February 2019, and post-COVID-19 assessments were conducted between March and June 2021; therefore, the assessment periods were not matched by calendar month. Seasonal and weather-related factors may have influenced physical activity and sedentary behavior. Given the small sample size and heterogeneous assessment periods, reliable adjustment for these factors was not feasible. Therefore, they should be considered potential sources of residual variability and confounding. Larger prospective studies with appropriate control groups and standardized accelerometry assessment periods are needed to confirm these preliminary observations.

## 5. Conclusions

Our findings indicate that patients with COPD managed in primary care had a predominantly sedentary physical activity profile at the pre-COVID-19 assessment. Among the seven participants with paired assessments, daily physical activity was lower at the post-COVID-19 assessment, particularly in terms of daily steps, while sedentary time increased. Exploratory analyses suggested inverse associations between physical activity and respiratory symptom burden, whereas associations with health-related quality of life varied according to the measure and assessment period. Psychological status, particularly anxiety, showed a less favorable profile at the post-COVID-19 assessment, while depressive symptoms remained relatively stable. Overall, these preliminary findings highlight the importance of considering physical activity, sedentary behavior, psychological well-being, and perceived health status as complementary aspects of COPD management. Given the small, paired sample and observational design, these findings should be considered exploratory and hypothesis-generating. Larger controlled longitudinal studies are needed to confirm these observations and clarify the factors underlying changes in physical activity, psychological status, and health-related quality of life.

## Figures and Tables

**Figure 1:**
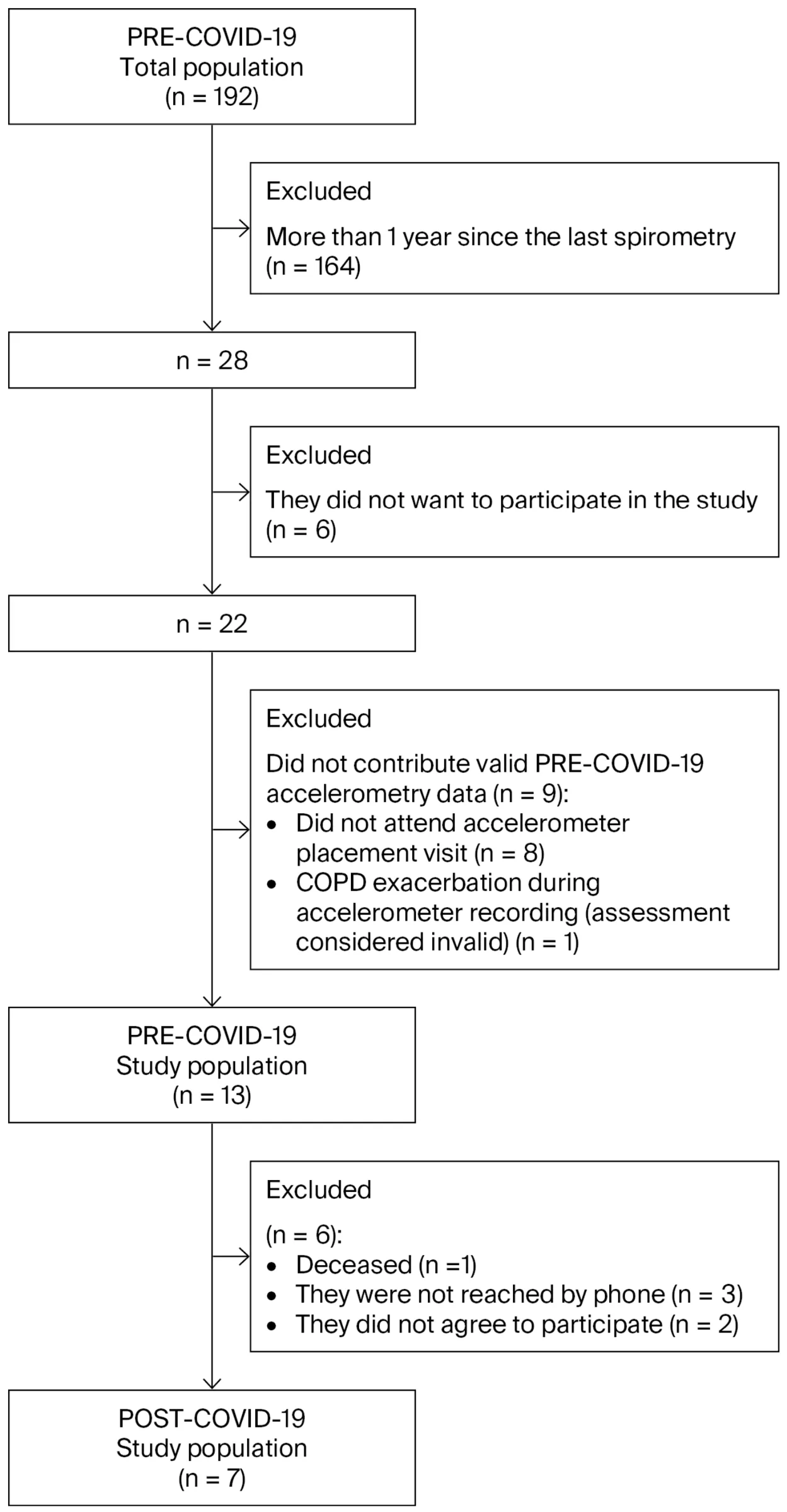
Flowchart of patient inclusion.

**Figure 2:**
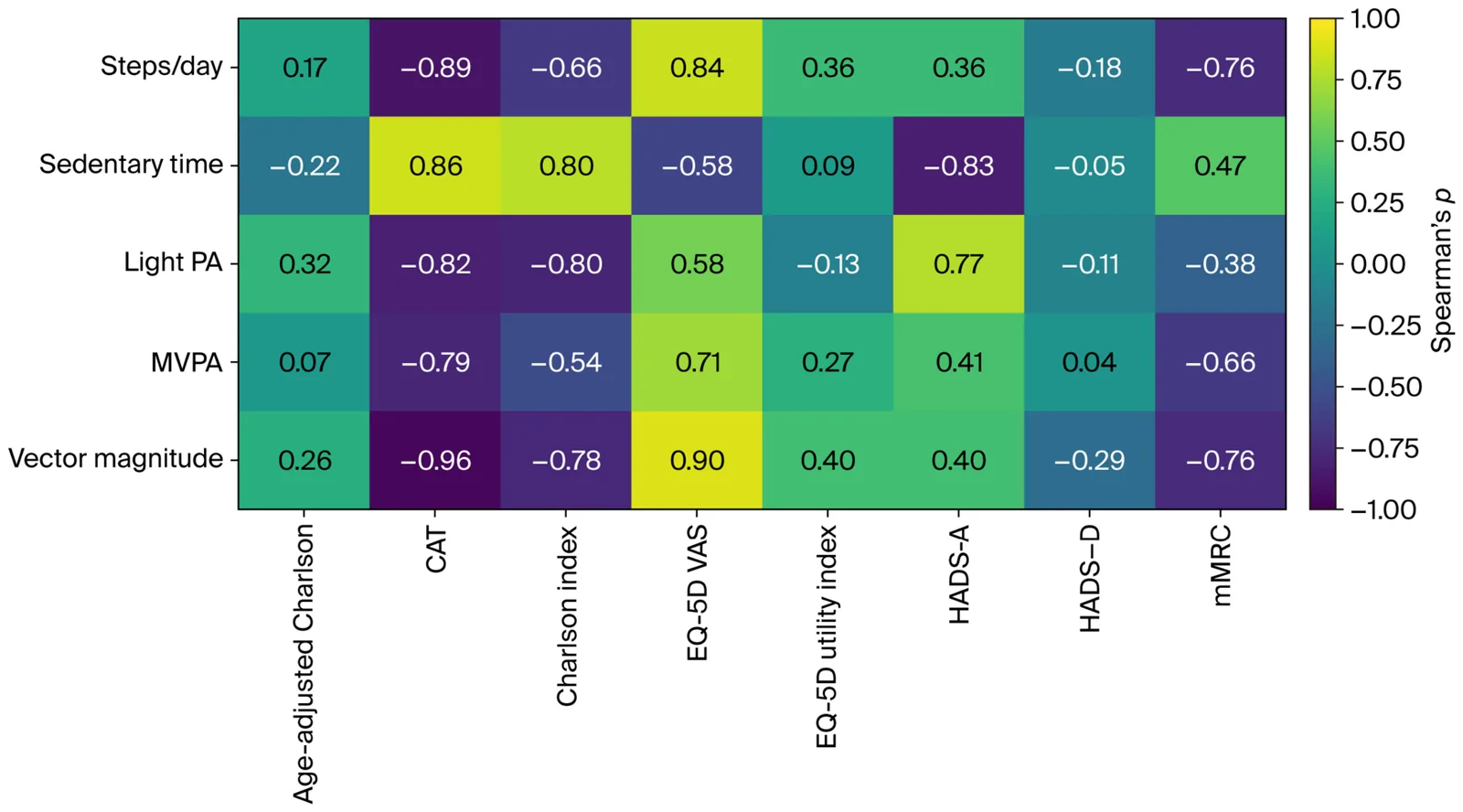
Exploratory Spearman correlation analysis between physical activity measures, clinical, health-related quality of life, and psychological variables at the pre-COVID-19 assessment in participants with paired longitudinal data (*n* = 7). Associations should be interpreted cautiously given the small sample size.

**Figure 3:**
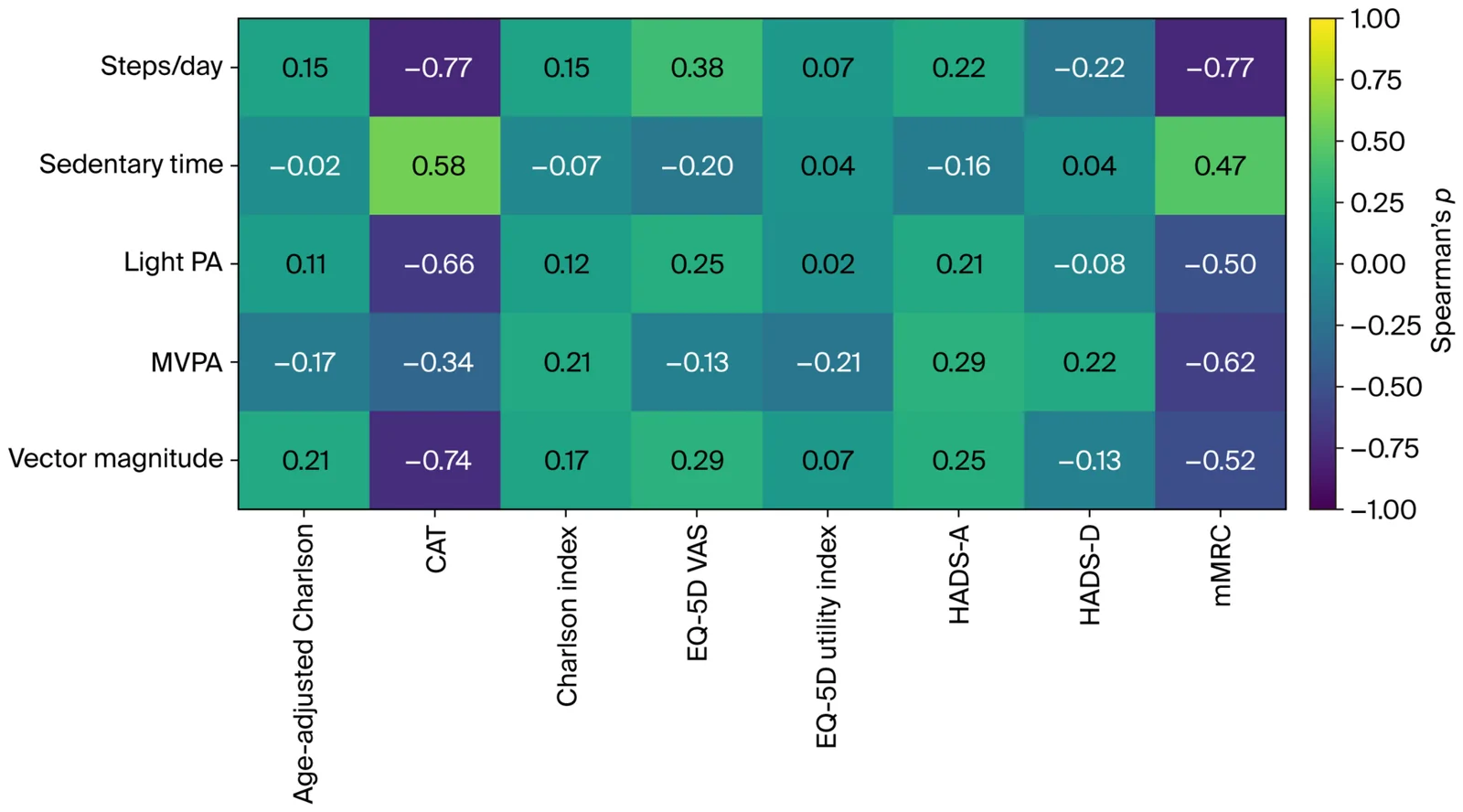
Exploratory Spearman correlation analysis between physical activity measures, clinical, health-related quality of life, and psychological variables at the post-COVID-19 assessment in participants with paired longitudinal data (*n* = 7). Associations should be interpreted cautiously given the small sample size.

**Table 1:** Demographic and clinical characteristics of the pre-COVID-19 cohort and the paired longitudinal cohort *.

	Pre-COVID-19 Assessment	Paired Pre-COVID-19 Assessment	Paired Post-COVID-19 Assessment
Sample Size	*n* = 13	*n* = 7	*n* = 7
Frequency	Frequency, *n* (%)	Frequency, *n* (%)	Frequency, *n* (%)
Sex			
Women	1 (7.69%)	1 (14.28%)	1 (14.28%)
Men	12 (92.31%)	6 (85.72%)	6 (85.72%)
Age (years), mean ± SD (range)	67.47 ± 9.66 (53–91)	70.51 ± 11.23 (57–91)	72.73 ± 11.22 (60–93)
Living with others			
Yes	11 (84.61%)	5 (71.42%)	6 (85.71%)
No	2 (15.38%)	2 (28.57%)	1 (14.29%)
Smoking habits			
Active smoker	6 (46.15%)	4 (57.14%)	3 (42.85%)
Ex-smoker	7 (53.84%)	3 (42.85%)	4 (57.14%)
Degree of obstruction: FEV1, % predicted (mean ± SD (min–max))	55.07 ± 18.88 (13–90)	47.28 ± 22.86 (13–90)	NA ^†^
Mild (≥80%)	1 (7.69%)	1 (14.28%)	NA ^†^
Moderate (50–79%)	7 (53.84%)	1 (14.28%)	NA ^†^
Severe (30–49%)	4 (30.76%)	4 (57.14%)	NA ^†^
Very severe (<30%)	1 (7.69%)	1 (14.28%)	NA ^†^
≥2 Moderate/severe exacerbations	6 (46.15%)	2 (28.57%)	1 (14.28%)
Employment			
Active	2 (15.38%)	1 (14.28%)	0
Disabled/pre-retired	2 (15.38%)	1 (14.28%)	2 (28.57%)
Retired	9 (69.23%)	5 (71.42%)	5 (71.42%)
BMI, mean ± SD (range)	30.25 ± 6.27 (20.69–43.65)	28.14 ± 4.43 (20.69–34.8)	29.03 ± 4.69 (22.6–34.56)
Normal weight (18.5–24.9)	1 (7.69%)	1 (14.28%)	2 (28.57%)
Pre-obesity (25–29.9)	7 (53.84%)	3 (42.85%)	2 (28.57%)
Obesity grade 1 (30–34.9)	3 (23.07%)	2 (28.57%)	3 (42.85%)
Obesity grade 2 (35–39.9)	0	0	0
Obesity grade 3 (≥40)	2 (15.38%)	0	0
Bronchodilator treatment			
No treatment	1 (7.69%)	0	0
LABA or LAMA	4 (30.76%)	4 (57.14%)	3 (42.85%)
LABA + LAMA	4 (30.76%)	1 (14.28%)	1 (14.28%)
LABA or LAMA + ICS	1 (7.69%)	0	0
LABA + LAMA + ICS	2 (15.38%)	1 (14.28%)	2 (28.57%)
SABA/SAMA	1 (7.69%)	1 (14.28%)	1 (14.28%)
Supplemental oxygen			
Yes	1 (7.69%)	1 (14.28%)	1 (14.28%)
No	12 (92.30%)	6 (85.71%)	6 (85.71%)
Charlson Comorbidity Index	1.84 ± 0.80 (1–2)	2.14 ± 0.69 (1–3)	2.14 ± 1.06 (1–4)
Age-adjusted Charlson Index	4.23 ± 1.09 (3–7)	4.71 ± 1.25 (3–7)	5.14 ± 1.21 (4–6)

* Data are presented as the mean ± SD (range) for continuous variables and *n* (%) for categorical variables. The paired columns include only participants with available data at both pre-COVID-19 and post-COVID-19 assessments (*n* = 7). Percentages in the paired cohort should be interpreted in the context of the small sample size. ^†^ Spirometry was not repeated at the post-COVID-19 assessment; therefore, no longitudinal change in FEV1 could be evaluated.

**Table 2:** Clinical indices in the pre- and post-COVID-19 populations.

	Pre-COVID-19 Assessment	Paired Pre-COVID-19 Assessment	Paired Post-COVID-19 Assessment
Sample Size	*n* = 13	*n* = 7	*n* = 7
Frequency	Frequency, *n* (%)	Frequency, *n* (%)	Frequency, *n* (%)
Degree of impact: CAT (mean ± SD (min–max))	12.23 ± 8.58 (1–30)	13.42 ± 10.58 (1–30)	13.57 ± 10.03 (0–25)
Low impact (1–10 points)	6 (46.15%)	3 (42.85%)	3 (42.85%)
Medium impact (11–20 points)	5 (38.46%)	2 (28.57%)	1 (14.28%)
High impact (21–30 points)	1 (7.69%)	1 (14.28%)	3 (42.85%)
Very high impact (31–40 points)	1 (7.69%)	1 (14.28%)	0
Dyspnea: mMRC (mean ± SD (min–max))	0.76 ± 0.72 (0–2)	1 ± 0.81 (0–2)	1.57 ± 1.27 (0–4)
mMRC 0	5 (38.46%)	2 (28.57%)	1 (14.28%)
mMRC 1	6 (46.15%)	3 (42.85%)	3 (42.85%)
mMRC 2	2 (15.38%)	2 (28.57%)	2 (28.57%)
mMRC 3	0	0	0
mMRC 4	0	0	1 (14.28%)
GOLD 2017			
A	4 (30.76%)	3 (42.85%)	2 (28.57%)
B	3 (23.07%)	2 (28.57%)	4 (57.14%)
C	1 (7.69%)	0	1 (14.29%)
D	5 (38.46%)	2 (28.57%)	0
GOLD 2025			
A	4 (30.76%)	3 (42.85%)	2 (28.57%)
B	3 (23.07%)	2 (28.57%)	4 (57.14%)
E	6 (46.15%)	2 (28.57%)	1 (14.29%)
EQ-5D index			
EQ-5D utility index (mean ± SD)	0.712 ± 0.308	0.749 ± 0.207	0.642 ± 0.216
EQ-5D VAS total (mean ± SD (min–max))	65 ± 11.90 (30–85)	60 ± 22.36 (20–80)	57.14 ± 19.97 (30–85)
EQ-5D affected dimensions			
Mobility problems total, *n* (%)	5 (38.46)	3 (42.85)	6 (85.71)
Self-care problems total, *n* (%)	2 (15.38)	1 (14.28)	2 (28.57)
Usual activities problems total, *n* (%)	4 (30.77)	2 (28.57)	1 (14.29)
Pain/discomfort problems total, *n* (%)	5 (38.46)	2 (28.57)	4 (57.14)
Anxiety/depression problems total, *n* (%)	8 (61.54)	4 (57.14)	5 (71.43)

**Table 3:** Longitudinal changes in objectively measured physical activity among participants with paired pre- and post-COVID-19 assessments (*n* = 7) *.

Variable	Pre-COVID-19 Assessment (Mean ± SD (min–max))	Pre-COVID-19 Median (IQR)	Post-COVID-19 Assessment (Mean ± SD (min–max))	Post-COVID-19 Median (IQR)	Median Paired Change (95% CI)	*p*-Value	Effect Size
Sedentary time (%)	82.07 ± 6.38 (72.40–87.90)	85.63 (77.89–86.38)	85.66 ± 9.19 (65.80–92.90)	88.7 (85.55–90.55)	2.4 (0.37 to 7.27)	0.219	0.57
Low-intensity physical activity (%)	15.92 ± 4.67 (11.56–23.99)	13.93 (12.38–18.60)	13.17 ± 8.16 (6.60–30.90)	10.4 (9.30–12.85)	−2.80 (−7.33 to −0.16)	0.219	−0.57
Moderate-to-vigorous physical activity (%)	2.01 ± 2.20 (0.10–6.20)	1.02 (0.57–2.82)	1.19 ± 1.13 (0.10–3.30)	0.9 (0.35–1.65)	−0.12 (−2.90 to 0.10)	0.375	−0.43
Vector magnitude (CPM)	286.89 ± 144.42 (150.00–576.70)	218.1 (209.45–322.25)	312.61 ± 249.45 (157.80–866.20)	249.6 (179.90–277.45)	11.9 (−46.30 to 76.80)	0.813	0.14
Steps/day	6179.82 ± 3862.65 (2031–11,740.29)	5348.43 (3474.50–8595.00)	3038.05 ± 1914.70 (1225.63–6258.75)	2584.63 (1432.31–4166.38)	−2272.71 (−5481.54 to −805.38)	0.031	−0.93

* Data are presented as mean ± SD and median (IQR). Paired change represents the median of individual differences for post- minus pre-assessments. The 95% confidence intervals for paired changes were estimated using bootstrap resampling. *p*-values were obtained using the exact Wilcoxon signed-rank test, and effect sizes are reported as rank–biserial correlations. Given the small, paired sample (*n* = 7), all longitudinal comparisons should be considered exploratory. Abbreviations: CPM, counts per minute; SD, standard deviation; IQR, interquartile range; CI, confidence interval.

**Table 4:** Steps/day and activity clusters in the seven paired participants.

Patient	Steps/Day PRE	Physical Activity Category PRE	Steps/Day POST	Physical Activity Category POST	Change (Pre–Post)
1	4150.00	Sedentary	4606.75	Sedentary	+456.75
2	2031.00	Sedentary	1225.63	Sedentary	−805.37
3	11,191.29	Active	1577.63	Sedentary	−9613.66
4	5998.71	Low active	3726.00	Sedentary	−2272.71
5	2799.00	Sedentary	1287.00	Sedentary	−1512.00
6	11,740.29	Active	6258.75	Low active	−5481.54
7	5348.43	Low active	2584.63	Sedentary	−2763.80

**Table 5:** Psychological status assessed using the Hospital Anxiety and Depression Scale (HADS) in the paired pre- and post-COVID-19 cohort (*n* = 7).

Variable	Pre-COVID-19 Assessment *n* (%)	Post-COVID-19 Assessment *n* (%)
HADS total (mean ± SD)	11.71 ± 8.63	13.43 ± 10.49
HADS total, *n* (%)		
<8 (normal)	2 (28.6%)	3 (42.9%)
8–10 (borderline)	2 (28.6%)	1 (14.3%)
≥11 (clinical)	3 (42.9%)	3 (42.9%)
HADS-A (mean ± SD)	5.14 ± 5.17	7.57 ± 5.19
Normal (0–7)	5 (71.4%)	3 (42.9%)
Borderline (8–10)	0 (0%)	2 (28.6%)
Clinical (≥11)	2 (28.6%)	2 (28.6%)
HADS-D (mean ± SD)	6.6 ± 5.2	5.86 ± 6.34
Normal (0–7)	5 (71.4%)	6 (85.7%)
Borderline (8–10)	0 (0%)	0 (0%)
Clinical (≥11)	2 (28.6%)	1 (14.3%)

## Data Availability

The datasets used and/or analyzed during the current study are available from the corresponding author on reasonable request. The datasets generated and/or analyzed during this study are not publicly available due to ethical and regulatory restrictions related to the protection of participants’ privacy.

## Supplementary Materials

The following supporting information can be downloaded at: https://www.mdpi.com/article/10.3390/healthcare14193142/s1, Table S1. Baseline characteristics of participants who completed longitudinal follow-up and those who did not. Table S2. Individual GOLD classification at PRE- and POST-COVID-19 assessments among the seven paired participants. Figure S1. Individual trajectories of physical activity measures between PRE- and POST-COVID-19 assessments in the paired cohort (*n* = 7). File S1: Age-modified Charlson Comorbidity Index. File S2: Modified Medical Research Council (mMRC) scale. File S3: COPD Assessment Test (CAT). File S4: HAD QUESTIONNAIRE: Hospital anxiety and depression. File S5: QUESTIONNAIRE EQ-5D.

## Abbreviations

The following abbreviations are used in this manuscript:

ABCD	Assessment Tool Based on Symptoms and Exacerbation Risk according to the GOLD 2017 Classification
ABE	Assessment Tool Based on Symptoms and Exacerbation Risk According to the GOLD 2023/2025 Classification
AECOPD	Acute Exacerbation of Chronic Obstructive Pulmonary Disease
CAT	COPD Assessment Test
CCI	Charlson Comorbidity Index
COPD	Chronic Obstructive Pulmonary Disease
CPM	Counts Per Minute
COVID-19	Coronavirus Disease 2019
EQ-5D	EuroQol Five-Dimension Questionnaire
FEV_1_	Forced Expiratory Volume in One Second
FEV_1_/FVC	Ratio of Forced Expiratory Volume in One Second to Forced Vital Capacity
GOLD	Global Initiative for Chronic Obstructive Lung Disease
GOLD 2017	2017 Global Initiative for Chronic Obstructive Lung Disease Report
GOLD 2025	2025 Global Initiative for Chronic Obstructive Lung Disease Report
HADS	Hospital Anxiety and Depression Scale
ICS	Inhaled Corticosteroids
LABA	Long-Acting β_2_-Agonist
LAMA	Long-Acting Muscarinic Antagonist
mMRC	Modified Medical Research Council Dyspnoea Scale
PA	Physical Activity
PC	Primary Care
Post-COVID-19	Follow-up Assessment Period (2021)
Pre-COVID-19	Baseline Assessment Period (2019)
SABA	Short-Acting β_2_-Agonist
SAMA	Short-Acting Muscarinic Antagonist
SD	Standard Deviation
USD	United States Dollars
VM	Vector Magnitude

## References

[1] Global Initiative for Chronic Obstructive Lung Disease (2025). Global Strategy for the Prevention, Diagnosis, and Management of Chronic Obstructive Pulmonary Disease: 2025 Report.

[2] Garcia-Aymerich J., Lange P., Benet M., Schnohr P., Antó J.M. (2006). Regular physical activity reduces hospital admission and mortality in chronic obstructive pulmonary disease: A population based cohort study. Thorax.

[3] Donaire-Gonzalez D., Gimeno-Santos E., Balcells E., de Batlle J., Ramon M.A., Rodriguez E., Farrero E., Benet M., Guerra S., Sauleda J. (2015). Benefits of physical activity on COPD hospitalisation depend on intensity. Eur. Respir. J..

[4] Moreno A., Montón C., Belmonte Y., Gallego M., Pomares X., Real J. (2009). Causas de muerte en pacientes con EPOC grave. Factores pronósticos [Causes of death and risk factors for mortality in patients with severe chronic obstructive pulmonary disease]. Arch. Bronconeumol..

[5] Waschki B., Kirsten A., Holz O., Müller K.C., Meyer T., Watz H., Magnussen H. (2011). Physical activity is the strongest predictor of all-cause mortality in patients with COPD: A prospective cohort study. Chest.

[6] Tang C.Y., Bernstein B., Blackstock F., Blondeel A., Gershon A., Gimeno-Santos E., Gloeckl R., Marques A., Spruit M.A., Garvey C. (2024). Unravelling the complex interplay of factors behind exercise limitations and physical inactivity in COPD. Breathe.

[7] Fiorentino G., Esquinas A.M., Annunziata A. (2020). Exercise and Chronic Obstructive Pulmonary Disease (COPD). Adv. Exp. Med. Biol..

[8] O’Donnell D.E., James M.D., Milne K.M., Neder J.A. (2019). The Pathophysiology of Dyspnea and Exercise Intolerance in Chronic Obstructive Pulmonary Disease. Clin. Chest Med..

[9] Pepin V., Saey D., Laviolette L., Maltais F. (2007). Exercise capacity in chronic obstructive pulmonary disease: Mechanisms of limitation. COPD J. Chronic Obstr. Pulm. Dis..

[10] Pitta F., Troosters T., Spruit M.A., Probst V.S., Decramer M., Gosselink R. (2005). Characteristics of physical activities in daily life in chronic obstructive pulmonary disease. Am. J. Respir. Crit. Care Med..

[11] Garcia-Aymerich J., Félez M.A., Escarrabill J., Marrades R.M., Morera J., Elosua R., Antó J.M. (2004). Physical activity and its determinants in severe chronic obstructive pulmonary disease. Med. Sci. Sports Exerc.

[12] Shu C.C., Lee J.H., Tsai M.K., Su T.C., Wen C.P. (2021). The ability of physical activity in reducing mortality risks and cardiovascular loading and in extending life expectancy in patients with COPD. Sci. Rep..

[13] Ramos M., Lamotte M., Gerlier L., Svangren P., Miquel-Cases A., Haughney J. (2019). Cost-effectiveness of physical activity in the management of COPD patients in the UK. Int. J. Chron. Obstruct Pulmon Dis..

[14] Nici L., Mammen M.J., Charbek E., Alexander P.E., Au D.H., Boyd C.M., Criner G.J., Donaldson G.C., Dreher M., Fan V.S. (2020). Pharmacologic Management of Chronic Obstructive Pulmonary Disease. An Official American Thoracic Society Clinical Practice Guideline. Am. J. Respir. Crit. Care Med..

[15] Cosío B.G., Hernández C., Chiner E., Gimeno-Santos E., Pleguezuelos E., Seijas N., Rigau D., López-Campos J.L., Soler-Cataluña J.J., Calle M. (2022). En nombre del equipo de trabajo de GesEPOC 2021. Spanish COPD Guidelines (GesEPOC 2021): Non-pharmacological Treatment Update. Arch. Bronconeumol..

[16] World Health Organization (2020). WHO Director-General’s Opening Remarks at the Media Briefing on COVID-19—11 March 2020 [Internet].

[17] Pépin J.L., Bruno R.M., Yang R.Y., Vercamer V., Jouhaud P., Escourrou P., Boutouyrie P. (2020). Wearable Activity Trackers for Monitoring Adherence to Home Confinement During the COVID-19 Pandemic Worldwide: Data Aggregation and Analysis. J. Med. Internet Res..

[18] Hume E., Armstrong M., Manifield J., McNeillie L., Chambers F., Wakenshaw L., Burns G., Heslop Marshall K., Vogiatzis I. (2020). Impact of COVID-19 shielding on physical activity and quality of life in patients with COPD. Breathe.

[19] McAuley H., Hadley K., Elneima O., Brightling C.E., Evans R.A., Steiner M.C., Greening N.J. (2021). COPD in the time of COVID-19: An analysis of acute exacerbations and reported behavioural changes in patients with COPD. ERJ Open Res..

[20] Charlson M.E., Pompei P., Ales K.L., MacKenzie C.R. (1987). A new method of classifying prognostic comorbidity in longitudinal studies: Development and validation. J. Chronic Dis..

[21] Bestall J.C., Paul E.A., Garrod R., Garnham R., Jones P.W., Wedzicha J.A. (1999). Usefulness of the Medical Research Council (MRC) dyspnoea scale as a measure of disability in patients with chronic obstructive pulmonary disease. Thorax.

[22] Jones P.W., Harding G., Berry P., Wiklund I., Chen W.H., Kline Leidy N. (2009). Development and first validation of the COPD Assessment Test. Eur. Respir. J..

[23] Quintana J.M., Padierna A., Esteban C., Arostegui I., Bilbao A., Ruiz I. (2003). Evaluation of the psychometric characteristics of the spanish version of the Hospital Anxiety and Depression Scale. Acta Psychiatr. Scand..

[24] Herrero M.J., Blanch J., Peri J.M., De Pablo J., Pintor L., Bulbena A. (2003). A validation study of the hospital anxiety and depression scale (HADS) in a Spanish population. Gen. Hosp. Psychiatry.

[25] Badia X., Roset M., Montserrat S., Herdman M., Segura A. (1999). The Spanish version of EuroQol: A description and its ap-plications. European Quality of Life scale. Med. Clin..

[26] Vogelmeier C.F., Criner G.J., Martinez F.J., Anzueto A., Barnes P.J., Bourbeau J., Celli B.R., Chen R., Decramer M., Fabbri L.M. (2017). Global Strategy for the Diagnosis, Management, and Prevention of Chronic Obstructive Lung Disease 2017 Report: GOLD Executive Summary. Eur. Respir. J..

[27] Troiano R.P., Berrigan D., Dodd K.W., Mâsse L.C., Tilert T., McDowell M. (2008). Physical activity in the United States measured by accelerometer. Med. Sci. Sports Exerc..

[28] Trost S.G., McIver K.L., Pate R.R. (2005). Conducting accelerometer-based activity assessments in field-based research. Med. Sci. Sports Exerc..

[29] Tudor-Locke C., Bassett J. (2004). How many steps/day are enough? Preliminary pedometer indices for public health. Sports Med..

[30] Soriano J.B., Alfageme I., Miravitlles M., de Lucas P., Soler-Cataluña J.J., García-Río F., Casanova C., Rodríguez González-Moro J.M., Cosío B.G., Sánchez G. (2021). Prevalence and Determinants of COPD in Spain: EPISCAN II. Arch. Bronconeumol..

[31] Miravitlles M., Soriano J.B., García-Río F., Muñoz L., Duran-Tauleria E., Sanchez G., Sobradillo V., Ancochea J. (2009). Prevalence of COPD in Spain: Impact of undiagnosed COPD on quality of life and daily life activities. Thorax.

[32] Koreny M., Demeyer H., Benet M., Arbillaga-Etxarri A., Balcells E., Barberan-Garcia A., Gimeno-Santos E., Hopkinson N.S., De Jong C., Karlsson N. (2021). Patterns of Physical Activity Progression in Patients With COPD. Arch. Bronconeumol..

[33] Pitta F., Takaki M.Y., Oliveira N.H., Sant’anna T.J., Fontana A.D., Kovelis D., Camillo C.A., Probst V.S., Brunetto A.F. (2008). Relationship between pulmonary function and physical activity in daily life in patients with COPD. Respir. Med..

[34] Garcia-Rio F., Lores V., Mediano O., Rojo B., Hernanz A., López-Collazo E., Alvarez-Sala R. (2009). Daily physical activity in patients with chronic obstructive pulmonary disease is mainly associated with dynamic hyperinflation. Am. J. Respir. Crit. Care Med..

[35] Lahaije A.J., van Helvoort H.A., Dekhuijzen P.N., Vercoulen J.H., Heijdra Y.F. (2013). Resting and ADL-induced dynamic hyperinflation explain physical inactivity in COPD better than FEV1. Respir. Med..

[36] Barrecheguren M., Monteagudo M., Ferrer J., Borrell E., Llor C., Esquinas C., Miravitlles M. (2016). Treatment patterns in COPD patients newly diagnosed in primary care. A population-based study. Respir. Med..

[37] Spyratos D., Chloros D., Michalopoulou D., Tsiouprou I., Christoglou K., Sichletidis L. (2021). Under-diagnosis, false diagnosis and treatment of COPD in a selected population in Northern Greece. Eur. J. Gen. Pract..

[38] Price D., West D., Brusselle G., Gruffydd-Jones K., Jones R., Miravitlles M., Rossi A., Hutton C., Ashton V.L., Stewart R. (2014). Management of COPD in the UK primary-care setting: An analysis of real-life prescribing patterns. Int. J. Chron. Obstruct Pulmon Dis..

[39] Burge A.T., Cox N.S., Abramson M.J., Holland A.E. (2020). Interventions for promoting physical activity in people with chronic obstructive pulmonary disease (COPD). Cochrane Database Syst. Rev..

[40] Miravitlles M., García-Rivero J.L., Ribera X., Galera J., García A., Palomino R., Pomares X. (2022). Exercise capacity and physical activity in COPD patients treated with a LAMA/LABA combination: A systematic review and meta-analysis. Respir. Res..

[41] Tan J.Y., Conceicao E.P., Wee L.E., Sim X.Y.J., Venkatachalam I. (2021). COVID-19 public health measures: A reduction in hospital admissions for COPD exacerbations. Thorax.

[42] Chan K.P.F., Ma T.F., Kwok W.C., Leung J.K.C., Chiang K.Y., Ho J.C.M., Lam D.C.L., Tam T.C.C., Ip M.S.M., Ho P.L. (2020). Significant reduction in hospital admissions for acute exacerbation of chronic obstructive pulmonary disease in Hong Kong during coronavirus disease 2019 pandemic. Respir. Med..

[43] Berghaus T.M., Karschnia P., Haberl S., Schwaiblmair M. (2022). Disproportionate decline in admissions for exacerbated COPD during the COVID-19 pandemic. Respir. Med..

[44] Tsai L.L., Alison J.A., McKenzie D.K., McKeough Z.J. (2016). Physical activity levels improve following discharge in people admitted to hospital with an acute exacerbation of chronic obstructive pulmonary disease. Chron. Respir. Dis..

[45] Sánchez Castillo S., Smith L., Díaz Suárez A., López Sánchez G.F. (2020). Associations between Physical Activity and Comorbidities in People with COPD Residing in Spain: A Cross-Sectional Analysis. Int. J. Environ. Res. Public Health.

[46] Harding S., Richardson A., Glynn A., Hodgson L. (2024). Influencing factors of sedentary behaviour in people with chronic obstructive pulmonary disease: A systematic review. BMJ Open Respir. Res..

