# Supplementary material for: Changes in Physical Activity and Psychological Status in Patients with COPD Between Pre- and Post-COVID-19 Assessments: A Pilot Observational Longitudinal Cohort Study
Source: Healthcare (Basel). 2026 Sep 22;14(19):3142. doi: 10.3390/healthcare14193142 (PMC13665032; doi:10.3390/healthcare14193142)

File S1: Age-modified Charlson Comorbidity Index.

Age-Adjusted Charlson Comorbidity Index (ACCI)

Comorbidity	Points
Myocardial infarction	1
Congestive heart failure	1
Peripheral vascular disease	1
Cerebrovascular disease	1
Dementia	1
Chronic pulmonary disease	1
Connective tissue disease	1
Peptic ulcer disease	1
Mild liver disease	1
Diabetes mellitus without end-organ damage	1
Hemiplegia or paraplegia	2
Moderate to severe renal disease	2
Diabetes mellitus with end-organ damage	2
Solid tumor without metastasis	2
Leukemia	2
Lymphoma	2
Moderate to severe liver disease	3
Metastatic solid tumor	6
AIDS	6

Age Adjustment

Age (years)	Additional points
< 50	0
50–59	+1
60–69	+2
70–79	+3
80–89	+4
≥ 90	+5

File S2: Modified Medical Research Council (mMRC) scale.

Scale	Severity of dyspnoea
0	No breathlessness except with strenuous exercise
1	Shortness of breath when hurrying on the level or walking up a slight hill
2	Walks slower than people of the same age on the level because of breathlessness or has to stop for breath when walking at own pace on the level
3	Stops for breath after walking about 100 metres or after a few minutes on the level
4	Too breathless to leave the house or breathless when dressing or undressing

File S3: COPD Assessment Test (CAT)

How is your COPD? Take the COPD Assessment Test™ (CAT)

This questionnaire will help you and your healthcare professional measure the impact COPD (Chronic Obstructive Pulmonary Disease) is having on your wellbeing and daily life. Your answers, and test score, can be used by you and your healthcare professional to help improve the management of your COPD and get the greatest benefit from treatment.

For each item below, place a mark (X) in the box that best describes you currently. Be sure to only select one response for each question.

Example: I am very happy 0 X 2 3 4 5 I am very sad

			SCORE
I never cough	0 1 2 3 4 5	I cough all the time	
I have no phlegm (mucus) in my chest at all	0 1 2 3 4 5	My chest is completely full of phlegm (mucus)	
My chest does not feel tight at all	0 1 2 3 4 5	My chest feels very tight	
When I walk up a hill or one flight of stairs I am not breathless	0 1 2 3 4 5	When I walk up a hill or one flight of stairs I am very breathless	
I am not limited doing any activities at home	0 1 2 3 4 5	I am very limited doing activities at home	
I am confident leaving my home despite my lung condition	0 1 2 3 4 5	I am not at all confident leaving my home because of my lung condition	
I sleep soundly	0 1 2 3 4 5	I don't sleep soundly because of my lung condition	
I have lots of energy	0 1 2 3 4 5	I have no energy at all	
		**TOTAL SCORE**	

COPD Assessment Test and CAT logo is a trademark of the GlaxoSmithKline group of companies.
© 2009 GlaxoSmithKline. All rights reserved.

RES/QST/09/43163/1 Date of preparation: September 2009.

RESULTS:

**1-10 points:**	Low impact
**11-20 points:**	Medium impact
**21-30 points:**	High impact
**>30 points:**	Very high impact

File S4: HAD QUESTIONNAIRE: Hospital anxiety and depression

Hospital Anxiety and Depression Scale (HADS)

Tick the box beside the reply that is closest to how you have been feeling in the past week.
Don't take too long over you replies: your immediate is best.

D	A		D	A	
		**I feel tense or 'wound up':**			**I feel as if I am slowed down:**
	3	Most of the time	3		Nearly all the time
	2	A lot of the time	2		Very often
	1	From time to time, occasionally	1		Sometimes
	0	Not at all	0		Not at all
		**I still enjoy the things I used to enjoy:**			**I get a sort of frightened feeling like 'butterflies' in the stomach:**
0		Definitely as much		0	Not at all
1		Not quite so much		1	Occasionally
2		Only a little		2	Quite Often
3		Hardly at all		3	Very Often
		**I get a sort of frightened feeling as if something awful is about to happen:**			**I have lost interest in my appearance:**
	3	Very definitely and quite badly	3		Definitely
	2	Yes, but not too badly	2		I don't take as much care as I should
	1	A little, but it doesn't worry me	1		I may not take quite as much care
	0	Not at all	0		I take just as much care as ever
		**I can laugh and see the funny side of things:**			**I feel restless as I have to be on the move:**
0		As much as I always could		3	Very much indeed
1		Not quite so much now		2	Quite a lot
2		Definitely not so much now		1	Not very much
3		Not at all		0	Not at all
		**Worrying thoughts go through my mind:**			**I look forward with enjoyment to things:**
	3	A great deal of the time	0		As much as I ever did
	2	A lot of the time	1		Rather less than I used to
	1	From time to time, but not too often	2		Definitely less than I used to
	0	Only occasionally	3		Hardly at all
		**I feel cheerful:**			**I get sudden feelings of panic:**
3		Not at all		3	Very often indeed
2		Not often		2	Quite often
1		Sometimes		1	Not very often
0		Most of the time		0	Not at all
		**I can sit at ease and feel relaxed:**			**I can enjoy a good book or radio or TV program:**
	0	Definitely	0		Often
	1	Usually	1		Sometimes
	2	Not Often	2		Not often
	3	Not at all	3		Very seldom

Please check you have answered all the questions

Results:

0-7 points: Does not indicate a case.

8-10 points: Borderline case.

≥ 11 points: Probably, with cases in each of the subscales.

File S5: QUESTIONNAIRE EQ-5D

By placing a tick in one box in each group below, please indicate which statements best describe your own health state today

Mobility

I have no problems in walking about ☐
I have some problems in walking about ☐
I am confined to bed ☐

Self-Care

I have no problems with self-care ☐
I have some problems washing or dressing myself ☐
I am unable to wash or dress myself ☐

Usual Activities *(e.g. work, study, housework, family or leisure activities)*

I have no problems with performing my usual activities ☐
I have some problems with performing my usual activities ☐
I am unable to perform my usual activities ☐

Pain/Discomfort

I have no pain or discomfort ☐
I have moderate pain or discomfort ☐
I have extreme pain or discomfort ☐

Anxiety/Depression

I am not anxious or depressed ☐
I am moderately anxious or depressed ☐
I am extremely anxious or depressed ☐

Source: adapted from the EuroQol Group: www.euroqol.org

To help people say how good or bad a health state is, we have drawn a scale (rather like a thermometer) on which the best state you can imagine is marked 100 and the worst state you can imagine is marked 0.

We would like you to indicate on this scale how good or bad your own health is today, in your opinion. Please do this by drawing a line from the box below to whichever point on the scale indicates how good or bad your health state is today.

Your own
health state
today

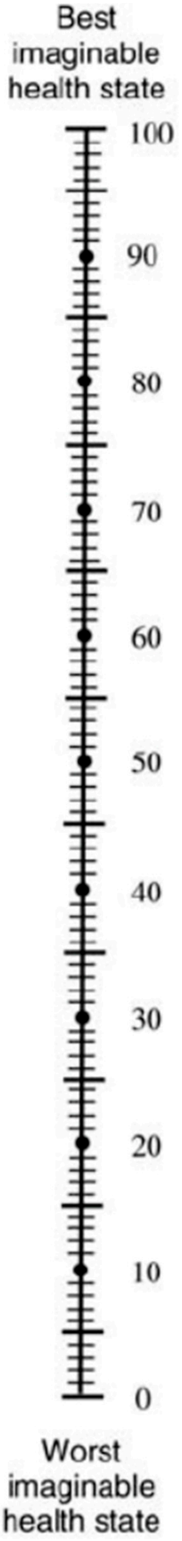

Supplement: Supplementary file 1 [file healthcare-14-03142-s001.zip › healthcare-4479648-supplementary Files.pdf]
