# Supplementary material for: Changes in Physical Activity and Psychological Status in Patients with COPD Between Pre- and Post-COVID-19 Assessments: A Pilot Observational Longitudinal Cohort Study
Source: Healthcare (Basel). 2026 Sep 22;14(19):3142. doi: 10.3390/healthcare14193142 (PMC13665032; doi:10.3390/healthcare14193142)
Supplement: Supplementary file 1 [file healthcare-14-03142-s001.zip › healthcare-4479648-supplementary Tables and Figure.pdf]

Table S1. Baseline characteristics of participants who completed longitudinal follow-up and those who did not.

Baseline Characteristic	Completers (*n* = 7)	Non-Completers (*n* = 6)
Age, years, mean ± SD	70.51 ± 11.23	63.67 ± 6.77
Sex, male, *n* (%)	6 (85.7%)	6 (100%)
FEV_1, % predicted, mean ± SD	47.29 ± 22.87	64.17 ± 6.68
mMRC score, mean ± SD	1.00 ± 0.82	0.50 ± 0.55
CAT score, mean ± SD	13.43 ± 10.58	10.83 ± 6.18
Charlson Comorbidity Index, mean ± SD	4.71 ± 1.25	3.67 ± 0.52
≥2 exacerbations in previous year, *n* (%)	2 (28.6%)	4 (66.7%)
GOLD E, *n* (%)	2 (28.6%)	4 (66.7%)
Steps/day, mean ± SD	6179.82 ± 3862.65	6278.74 ± 3064.07

Data are presented as mean ± SD or *n* (%), as appropriate. Comparisons between participants who completed longitudinal follow-up and those who did not are descriptive only. Given the very small group sizes (*n* = 7 and *n* = 6), no inferential statistical comparisons were performed. Abbreviations: FEV_1, forced expiratory volume in one second; mMRC, modified Medical Research Council; CAT, COPD Assessment Test; GOLD, Global Initiative for Chronic Obstructive Lung Disease; SD, standard deviation.

Table S2. Individual GOLD classification at PRE- and POST-COVID-19 assessments among the seven paired participants.

Patient	PRE-COVID-19 Gold 2017	PRE-COVID-19 Gold 2025	POST-COVID-19 Gold 2025
1	D	E	E
2	B	B	B
3	A	A	A
4	A	A	B
5	B	B	B
6	A	A	A
7	D	E	B

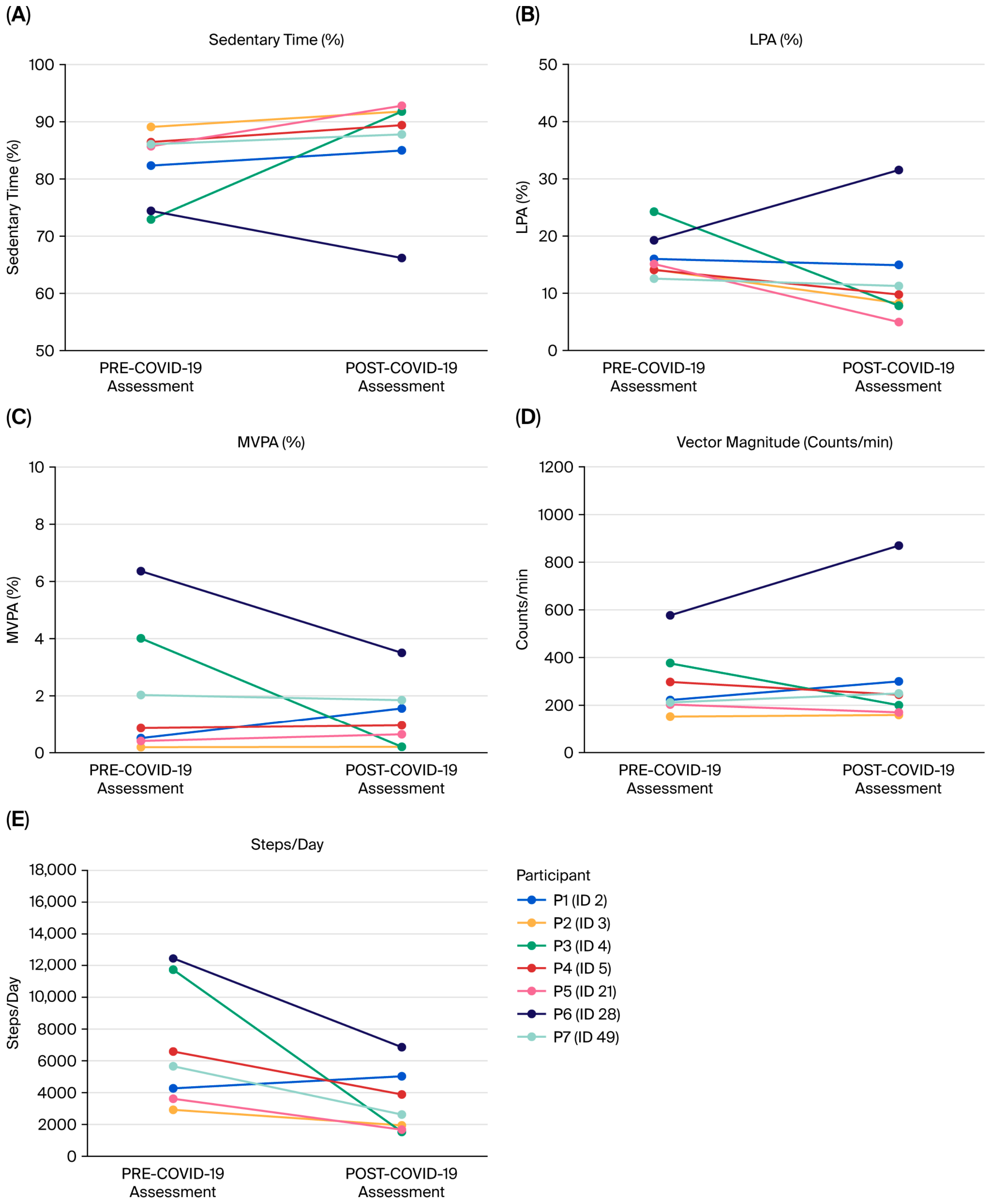

Figure S1. Individual trajectories of physical activity measures between PRE- and POST-COVID-19 assessments in the paired cohort (n = 7). Lines represent individual participants and show changes between assessments in sedentary time (%), light-intensity physical activity (LPA, %), moderate-to-vigorous physical activity (MVPA, %), vector magnitude (counts/min), and steps/day.
